# Dr. Walker's Remarks on Mr. Goldson's Pamphlet

**Published:** 1804-07-01

**Authors:** John Walker

**Affiliations:** Salisbury Square


### Dr. Walker's Remarks on Mr. Goldson's Pamphlet.

( Continued from Vol. xi. p. 505—508. )

THE insertion in your last Number of the Remarks, which I most hastily made on the cases of small-pox after supposed vaccination, require on my part a farther prosecution of the subject of Goldson's pamphlet, to which I now go to give an hour or two more.

Pages 36 to 40 exhibit two cases of small-pox produced thirteen and fourteen months after *(supposed)* "Vaccination by Mr. Weymouth." This gentleman had matter from the Central House on the 16th ult. *Allons.*

Page 41. "No difference in the appearance of the arm or the symptoms of Clark from any of the five marines." Then they will be all liable to take the small-pox as Clark and Sarah Smith did, if not re-inoculated.

Page 46. "It is not to be presumed that a public board, directing experiments to be made on the subject in an infirmary under their controul, would be so inattentive as to send such (matter) as was improper for that purpose. There could be no opportunity of its being decomposed, or deteriorated, while in possession of Mr. Rickman, as he used it on the same day he received it." But a stage coachman carrying in his pocket the little packet of guardian matter, getting off in the night to take his beer, and 'drawing up' to the fire of the inn to warm himself well before he again mounted his box, could decompose it. Matter, I believe from Golden Square, sealed up in great form, with the impression of a cow, I have known to prove effete; it was in the county of Surry. Matter packed with equal neatness, but heated in applying sealing wax, I have seen completely destroyed; it was on the Mediterranean between Gibraltar and Minorca.

Page 47. "From Langley (a marine) matter was taken for me, which I used on two children of Major Noel; and from these I vaccinated others in succession. They both resisted variolous inoculation six months afterwards." It sometimes happens to myself that I don't succeed in producing ducing the vaccine vesicle by the first puncture. Have the children, in such case, *resisted* the inoculation? It is perhaps through my extreme tenderness to them, scarcely letting them feel the lancet, or through some untoward application of the instrument, rather than from any sort of insusceptibility in the subject, that I have not succeeded. I have also failed for a time, at least in one instance, through the system being previously occupied with some other morbid affection. I can shew in this metropolis pretty permanent marks, though perhaps not indelible like the cicatrix or eschar invariably following vacciola, as well as *other* solutions of integument, produced on the skin by my vain attempts from week to week to vacciolate the subject. The child had extensive eruption both on the forehead and neck, the herpetic character of which manifested itself at the place of application of the lancet, and forbade the entrance of the vaccine virus into the system, while in the struggle, if I may so express it, *something like*, a *very little* like, cow-pox was produced. I was obliged to assure the mother that her child was not, and could not then be, protected. In less than three months after this I succeeded completely in vacciolating the child, and assured her that it was impervious to every attack of small-pox. The subject, as appears from the register, was Robert Hughes, No. 12, St. Paul's Chain, a child half a year old.

I have so often seen troublesome eruptions in children swept away by the vacciolous inoculation (the genuine vesicle being first produced from which there could be no objection to inoculate, but which in its last stages exhibited very exactly the light coloured scab of the previous eruption, instead of the dark one of uninterrupted vacciola) that I lately inoculated a child (completely vacciolated last year at the age of two months) because of its being very sore behind the ears. The mother in a week shewed her child much relieved, and having so specious a pock on its arm that a medical man so particular in the choice of his matter, that he waited at the Central House to see the subject it should be taken from, on arrival of this child preferred the specious pock, till he was informed of its history, when he contented himself with matter from one having more appearance of inflammation about it than was desirable. The subject was a child ten months old, Charles Good, No. 22, Lambeth Hill, Doctors' Commons.

Page 49. "Langley was found to resist variolous inoculation, &c." Suppose all this to be perfectly correct; it does does not follow that he resisted because of his having been previously inoculated. In Marmorice Harbour, on my endeavouring to set before some hesitating sailors, in the expedition to Egypt, the advantages of the vacciolous inoculation, some of them said they had often been exposed to small-pox without taking it. "So have I," said an old seaman, "till I was older than any of you. I have even helped in the hospital to carry out those that died of the small-pox, and escaped. But it caught me at last; you see how it has marked me, and I was half a year in recovering my strength after it." What a confirming case would this have been of W. G's conjectures, if the sailor had ever been previously, spuriously, vacciolated!

Does W. G. suppose that the Variolous Inoculation has never been incomplete? never spuriously conducted? The expedition, in Marmorice harbour, exhibited a motley assemblage from various nations; most of the numerous European languages were spoken in it; the Turkish, Arabic, &c. The errand of a figure, without uniform, daily setting out from the Admiral's ship, to visit the different vessels of the fleet, excited some attention.* I had instances mentioned to me of the Small-pox inoculation having failed to yield any protection in different quarters; but let me substantiate the general assertion by a particular statement. The lieutenant-colonel (Stuart) of the 42d (Highland) regiment, whose name appeared as an authority in the late dispute respecting the capture of the standard of the *Invincibles* before Alexandria, is very much marked with the small-pox. He told me he was inoculated when a boy, was considered to have gone thro' the disease; but afterwards took it in the natural way.

I am apprehensive that it may be supposed, from my holding the office of Resident Inoculator to the Royal Jennerian Society, that my remarks come from the Society itself, which the author has with such want of accuracy approached, by addressing his pamphlet "To the Directors of the *Vaccine Institution*." The proceedings of their Medical Council, read and approved at their last Quarterly Court, shew that they do not consider the pamphlet of W. G. in the same serious light that I do.

" At a Meeting of the Medical Council, May 15, 1804, Mr.

---

* Dr. Walker being at Malta when the expedition under Sir Ralph Abercrombie and Lord Keith arrived there, he consented to accompany it, the Small-pox having got into the fleet and being very fatal.

Mr. Ring in the chair, the following message was sent to the Board of Directors.

"The Medical Council having taken Mr. Goldson's Pamphlet into consideration, inform the Board of Directors, that they are of opinion it does not appear to require any particular notice from this Society."

Would it not be eligible and truly respectable for medical men in every quarter to form Jennerian Societies? It would distinctly mark the liberality and disinterestedness which so many of them, advocates for the new practice, already exercise in their profession. To do away every jealousy, let the oldest member of every association be the President; the youngest the Secretary. It belongs to them, in reality, the forming of the public opinion on medical affairs. Consider this ye guardians of public health! Ye have no idea of the exertions that are made by the gainsayers of the discovery, which, said an intelligent Greek to me in Egypt, is the '*Glory of your Nation,*' is the consolation of the world. Ye have no conception of the pains that are taken to spread terror and dismay among families, even by the gratuitous diffusion of the specious statements of the pamphlet under notice. Why did not the author, holding the copyright in his own hands, to prevent distraction among the unsuspecting multitudes, endeavour chiefly to confine his 'facts and observations' to your attention? Then they might have been examined "with that calmness and moderation which should ever accompany philosophical research." Had the manuscript been addressed to the Royal Jennerian Society, they would probably have considered all its errors as the exuberant effusions of an ingenuous though biassed mind, and paid a respectful attention to its mistaken and well meaning author. But no! it is committed, with all its faults, to the perusal of the wide world, to bewilder those who waver, to realarm those who were at length at rest. Hence the fond parents, unable to distinguish, may look on their blooming offspring, heretofore eyed with rapturous delight, in the saddest expectations, the most gloomy solicitude; and the lustre of the eye, which would beam on them consolation and joy, they may, by a tormenting anticipation, see closed up in endless night. But, surely, medical men will every where endeavour to dash the poison from the cup, and do justice to humanity, in preventing the weaker judgment of the credulous from being imposed on. Already the one or two remaining opposers of the new practice, in the profession, have failed in their attempts to produce small-pox after ter vacciolation, and the wealth and influence employed in giving gratuitous diffusion to the pernicious pamphlet will be deprived of their baneful and baleful effect while medical men remain unperverted. To them, I repeat it, belongs the forming of the public mind on medical affairs. The Royal Jennerian Society may long in vain continue to look towards public men, for *active* co-operation; they are all too much engaged with political affairs. If medical men, as was very early suggested by the President (Duke of Bedford) would come forward and co-operate with the Society, the great object would be attained, THE EXTERMINATION OF THE SMALL-POX.

I am, respectfully,

*Salisbury Square*, 14, *vj*. 1804. JOHN WALKER.

P. S. I have just been called upon by the gentleman that had the matter "for Dr. Waller, of Portsmouth," who wishes to know what were his exact words on application; and he has written for answer, that, "the exact words were a simple application for matter, with the remark that that which they have, *does not produce the desired effect*." In the mean time, applications from that quarter are continued at the Central House, as appears from the register.

"Mr. William Postlethwaite, Chichester."

"J. Wilkinson, Portsmouth."

"John Griffin, Minister of Orange Street Chapel, Portsea."

"William Munday, Chichester."

"Mr. Phillipson, Chichester."

The applicants from Portsmouth, like the thousands throughout the empire, as well as in both hemispheres, or on both sides of the Atlantic, and round the Capes Horn, Good Hope, and Van Diemen, are, the majority, unknown to me. These, with those whom I am personally acquainted with, must excuse my exhibition of their names. The great cause of humanity requires it; and when the ephemeral alarm from the pamphlet shall be quieted, the families they attend must recognise their seasonable vigilance, and, as I suppose, their *acumen* too, superior to that of the author, who with profession of honourable motives has, *perhaps unwittingly*, attempted a work with which that of Eratostratus the Ephesian, on the night of the birth of the founder of Alexandria, dwindles to a point without dimension in the comparison.

Will you now inform medical gentlemen, every where, through your extensively circulated Journal, that on application plication for vaccine ichor, they may save themselves the expence and trouble of paying the postage, by directing, "To the Secretary of the Royal Jennerian Society, Salisbury Square."

The increasing numbers of patients at the Central House, will, I hope, enable me to continue to supply every applicant. The Post Office has already liberally granted the privilege of sending it free throughout the empire; and it only requires some arrangements on the part of the Society to enable medical men to receive it without expence to themselves, and to co-operate in the great object of exterminating the Small-pox.

